# RETRACTION: Acute Pancreatitis Associated with the Antibiotic Levofloxacin: A Rare Case Report

**DOI:** 10.1002/ccr3.71702

**Published:** 2026-01-07

## Abstract

**RETRACTION**: InbanP. , GullaV. , DevaniA. , AkumaC. M. , GowthavaramC. A. , HussainA. , AungL. L. L. , JadavN. , SavaliyaB. , SajjadT. , KhanA. M. , AkumaO , KelechiA. E. , “Acute Pancreatitis Associated with the Antibiotic Levofloxacin: A Rare Case Report,” Clinical Case Reports 11, no. 12 (2023): e8186, 10.1002/ccr3.8186.38033693 PMC10682242

The above article, published online on 27 November 2023 in Wiley Online Library (wileyonlinelibrary.com), has been retracted by agreement between the journal Editor‐in‐Chief, Roberto Berebichez‐Fridman; and John Wiley & Sons, Ltd. The retraction has been agreed due to significant overlap found between this article and the following article published on 31 July 2023 in Cureus Journal of Medical Science, “A Rare Case of Levofloxacin‐Induced Acute Pancreatitis: Case Report and Literature Review” by O. Omoregie, U. T. Ugwuoke, O. Agho, U. C. Okonna, W. U. Iklaki, G. C. Okoro, *Cureus* 15, no. 7 (2023): e42743 (https://doi.org/10.7759/cureus.42743). The authors were contacted for comment but did not respond.

**RETRACTION**: P. Inban , V. Gulla , A. Devani , C. M. Akuma , C. A. Gowthavaram , A. Hussain , L. L. L. Aung , N. Jadav , B. Savaliya , T. Sajjad , A. M. Khan , O Akuma , A. E. Kelechi , “Acute Pancreatitis Associated with the Antibiotic Levofloxacin: A Rare Case Report,” Clinical Case Reports 11, no. 12 (2023): e8186, 10.1002/ccr3.8186.38033693 PMC10682242

The above article, published online on 27 November 2023 in Wiley Online Library (wileyonlinelibrary.com), has been retracted by agreement between the journal Editor‐in‐Chief, Roberto Berebichez‐Fridman; and John Wiley & Sons, Ltd. The retraction has been agreed due to significant overlap found between this article and the following article published on 31 July 2023 in Cureus Journal of Medical Science, “A Rare Case of Levofloxacin‐Induced Acute Pancreatitis: Case Report and Literature Review” by O. Omoregie, U. T. Ugwuoke, O. Agho, U. C. Okonna, W. U. Iklaki, G. C. Okoro, *Cureus* 15, no. 7 (2023): e42743 (https://doi.org/10.7759/cureus.42743). The authors were contacted for comment but did not respond.

